# Invasive infection due to *Saprochaete capitata* in a young patient with hematological malignancies

**DOI:** 10.1590/S1517-838246220120447

**Published:** 2015-06-01

**Authors:** Ana Maria Rabelo de Carvalho Parahym, Pedro José Rolim, Carolina Maria da Silva, Igor de Farias Domingos, Sarah Santos Gonçalves, Edinalva Pereira Leite, Vera Lúcia Lins de Morais, Danielle Patrícia Cerqueira Macêdo, Reginaldo Gonçalves de Lima, Rejane Pereira Neves

**Affiliations:** 1 Universidade Federal de Pernambuco, Departamento de Micologia, Centro de Ciências Biológicas, Universidade Federal de Pernambuco, Recife, PE, Brasil, Departamento de Micologia, Centro de Ciências Biológicas, Universidade Federal de Pernambuco, Recife, PE, Brazil.; 2 Universidade Federal de Pernambuco, Departamento de Ciências Farmacêuticas, Centro de Ciências da Saúde, Universidade Federal de Pernambuco, Recife, PE, Brasil, Departamento de Ciências Farmacêuticas, Centro de Ciências da Saúde, Universidade Federal de Pernambuco, Recife, PE, Brazil.; 3 Laboratório de Micologia Especial, São Paulo, SP, Brasil, Laboratório de Micologia Especial, São Paulo, SP, Brazil.; 4 Fundação Oswaldo Cruz, Instituto Oswaldo Cruz, Recife, PE, Brasil, Hospital Universitário do Instituto Oswaldo Cruz, Centro de Oncologia, Recife, PE, Brazil.

**Keywords:** invasive infection, *Saprochaete capitata*, *Geotrichum capitatum*, hematological malignancies, antifungal susceptibility

## Abstract

We report a case of invasive infection due to *Saprochaete capitata* in a patient with hematological malignancies after chemotherapy treatment and empiric antifungal therapy with caspofungin. Although severely immunocompromised the patient survived been treated with amphotericin B lipid complex associated with voriconazole.

*Saprochaete capita* is a saprophytic soil yeast (Pimentel *et al.*, 2005) and may rarely cause invasive systemic infections in immunocompromised patients (Schuermans *et al.*, 2012).

This is an emerging pathogen and fungal infections have been reported in patients with hematological malignancies (Girmenia *et al.*, 2005) and admitted to the intensive care unit (Gurgi *et al.*, 2011).

The treatment for *S. capitata* infections remains undetermined. However treatments with echinocandins antifungals generally are ineffective (Chittick *et al.*, 2009) and empirical treatment with these drugs may be is a predisposing factor for this yeast infections (Kubiak *et al.*, 2010).

A 15-year-old man with acute myeloid leukemia (AML) was admitted in the Pediatric Oncology Center, Oswaldo Cruz University Hospital, Recife, Brazil, for chemotherapy. The patient received induction therapy with cytosine arabinoside and mitoxantrone. Thereafter, was realized antibiotic prophylaxis with sulfamethoxazole-trimethoprim and antifungal prophylaxis with caspofungin (50 mg/day). On day 5 of hospitalization, the peripheral blood white cell count was 160/mm^3^ with an absolute neutrophil count of 10/mm^3^ and the platelet count was 8,000/mm^3^. On day 7 of hospitalization, the patient presented fever (38.5 °C), chills and abdominal pain. At that time the antimicrobial regimen was empirically changed by vancomycin, amikacin and imipenem-cilastatin sodium, and caspofungin was maintained. On day 8, ultrasound of the abdomen showed hepatosplenomegaly with splenic nodules and ureterohydronephrosis.

After a worsening of symptoms, the patient was admitted to the Intensive Care Unit of the same hospital. At the moment the patient showed respiratory insufficiency, septic shock, pyelonephritis, hematuria and acute renal failure. A computerized tomography of the chest showed pleural effusion in the right lung and pulmonary nodules in the left lung (Figure 1). Polymerase chain reaction was performed for tuberculosis but was negative. According to the clinical aspects of patient a probable systemic fungal infection with involvement of the spleen, kidney and lung was suspected and clinical samples were collected for mycological diagnosis.

**Figure 1:**
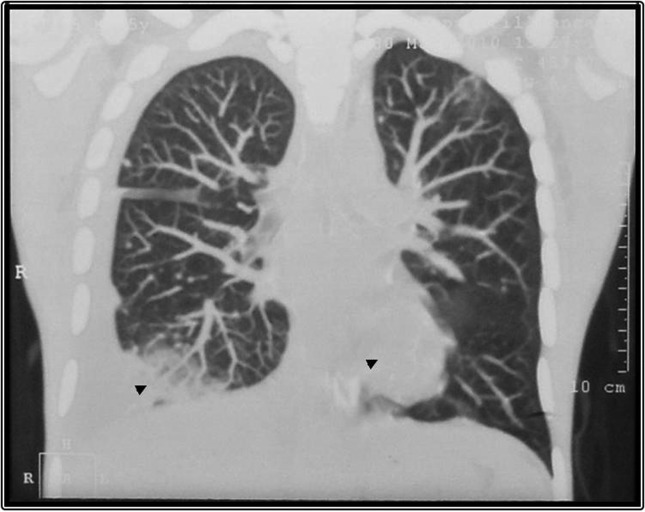
Computerized tomography chest showing pleural effusion in the right lung and pulmonary nodules in the left lung.

Samples of blood and urine were collected on three consecutive days. Venous blood samples were collected aseptically from the central and peripheral veins by venipuncture and urine specimens were collected in aseptic tubes after the urinary catheter is removed and performed asepsis in genital region. Because of severe thrombocytopenia, it was not possible to perform biopsies of the organs with abnormal ultrasound findings. All samples were processed immediately after collection by standard methods of mycological diagnosis.

Microbiological identification was achieved using traditional taxonomy through biochemical tests (assimilation of carbon and nitrogen), enzyme assay (urease), morphophysiological characteristics and by sequencing fragments of the internal transcribed spacer region of the rDNA using primers ITS-1 and ITS-4.

Antifungal susceptibility testing was performed in accordance with protocols defined by the Clinical and Laboratory Standards Institute (CLSI) M27-A3 (CLSI, 2008). The antifungal drugs amphotericin B (AMB), anidulafungin (ANI), fluconazole (FLZ) and voriconazole (VRZ) were evaluated. Quality control was performed by testing CLSI-recommended strains *Candida krusei* (ATCC 6528) and *C. parapsilosis* (ATCC 22019).

Direct examination of the urine samples showed septate hyaline hyphae and arthroconidia (Figure 2A) and in culture after seven days of growth at 30 and 37 °C on Sabouraud Dextrose Agar (Difco) media were visualized yeast-like fungi with colonies white to cream-colored, dry and wrinkled were seen in pure culture of all urine and blood samples. Microscopic analysis of the colony showed numerous arthroconidia and septated hyaline hyphae (Figure 2B).

**Figure 2:**
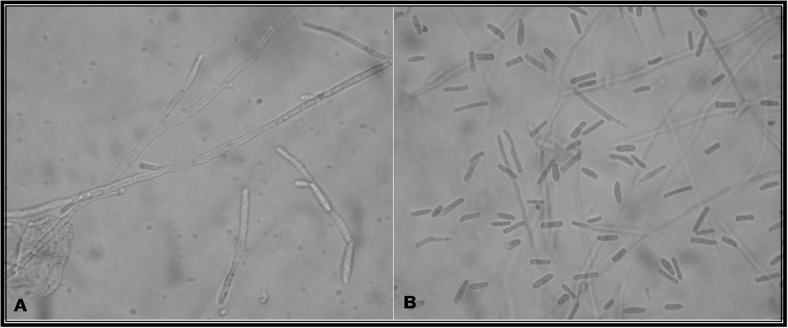
Arthroconidia and hyaline hyphae: direct examination (A) and Microscopic analysis of the culture performed from Sabouraud dextrose agar medium after 7 days incubation at 37 °C consistent with *Saprochaete capitata* (B).

The organism was negative for urea hydrolysis and for assimilation of D-xylose, D-arabinose, sucrose, lactose, Meα-D-glucoside, maltose, raffinose, soluble starch, trehalose, cellobiose, inullin and nitrate. The isolated only assimilated glucose and galactose. According to these morphological and biochemical characteristics this specimen was identified with *S. capitata.*

A BLAST search exhibited a 100% match to all the *Dipodascus capitatus* (*S. capitata* or *Magnusiomyces capitatus*) ITS sequences in the GenBank database.

The DNA sequence was submitted to GenBank with the accession number (JN573270). The isolate was submitted to a stock collection of Department of Mycology, Federal University of Pernambuco, Brazil, with record number 6260.

Minimal inhibitory concentrations for AMB, ANI, FLZ and VRZ were 0.06 μg/mL, 8 μg/mL, 32 μg/mL and 1 μg/mL, respectively. The isolated was sensitive to AMB and VRZ. Accordingly amphotericin B lipid complex (Abelcet®) (5 mg/kg/day) was administered intravenously for 24 days and after voriconazole (400 mg/day) (Pfizer Incorporated, New York, NY) orally. The patient was discharged and oral antifungal therapy was continued until neutrophils count greater than 500 mm^3^.

After attainment urine and blood cultures negative, remission from pulmonary, renal and splenic lesions, and the detection of normal sonographic findings, the patient was considered cured.

*S. capitata*, formerly known as *Geotrichum capitatum*, *Trichosporon capitatum* and *Blastoschizomyces capitatus* (teleomorph *D. capitatus*) was included in an taxonomic review based on the ribosomal structure and according to this new taxonomy was re-named *S. capitata* (teleomorph *M. capitatus*) (De Hoog and Smith, 2004).

Invasive systemic infections due to *S. capitata* have been reported in immunocompromised patients. Such infections can affect lung, liver, spleen, kidney, bone marrow, central nervous system and heart. However bloodstream infections are the most common clinical form (Schuermans *et al.*, 2011).

Two cases of fatal disseminated infection due this pathogen were reported associated with contaminated milk in hematological unit (Gurgi *et al.*, 2011).

In a retrospective multicenter study published in 2005 (Girmenia *et al.*, 2005) the authors described 35 cases of infection due to *S. capitata* diagnosed in period of 20 years in Italian patients with hematological diseases. From these 74.3% occurred in patients with AML and fungemia was diagnosed in 26 cases and only one case of probable tract urinary infection was documented by multiple urine cultures positive and sonographic evidence of renal lesions.

Other study describes a case of disseminated infection due *S. capitata* in an Australian patient with acute lymphoblastic leukemia. The authors detected branching and septate hyphae by microscopic examination of the kidneys, liver and spleen during *postmortem* examination and blood cultures consistent with this specie were obtained before death (Pimentel *et al.*, 2005).

In Brazilian patient with leukemia a fatal case of disseminated infection due this yeast was described. The patient was treated with conventional AMB (1 mg/kg/day) associated with itraconazole and after substituted for VRZ (Lafayette *et al.*, 2011). However, in our case the patient was cured using AMB lipid complex and after VRZ. According to our results AMB lipid complex associated with VRZ are the drugs of choice for the treatment of invasive infections by *S. capitata.*

*In vitro* assays conducted in another study indicated that *S. capitata* is susceptible to AMB, VRZ and flucytosine, and resistant to echinocandins and FLZ (as dependent dose) (Cuenca-Estrella *et al.*, 2006). In our study the isolate of *S. capitata* was not sensitive to echinocandin tested (ANI).

The optimal antifungal therapies for infections due to *S. capitata* remain unclear by the rarity of this etiologic agent and few studies. However treatments with echinocandins antifungals generally are ineffective (Chittick *et al.*, 2009) and 10.7% of the patients with this infection were treated empirically with caspofungin (Kubiak *et al.*, 2010).

Shuermans *et al.* (2011) described a case of invasive infection in leukemia patient after caspofungin treatment. In our report the patient was empirically treated with caspofungin and after showed clinical aspects of fungal infection being isolated *S. capitata* in urine and blood samples. Because the occurrence of thrombocytopenia was not possible to collect more invasive samples, however according to the clinical manifestations and sonographic evidence can be suggested a disseminated infection.

In summary, invasive infection by *S. capitata* occurs in patient with leukemia and treatment empirical with caspofungin is not effective and is probably a predisposing factor of this infection. Antifungal treatment realized with AMB lipid complex plus VRZ is effective against infections caused by *S. capitata*.
